# Comparing the financial, energy and time costs of different food and stove combinations in Nairobi using controlled cooking tests and longitudinal fuel price data

**DOI:** 10.14324/111.444/ucloe.3034

**Published:** 2025-03-26

**Authors:** Tash Perros, Mark O’Keefe, James Mwitari, Lewis Gichane, Elisa Puzzolo, Daniel Pope

**Affiliations:** 1 Department of Public Health, Policy and Systems, University of Liverpool, Liverpool, UK; 2 UCL Institute for Sustainable Resources, University College London, London, UK; 3 Sun King, Nairobi, Kenya; 4 Centre for Respiratory Diseases Research, Kenya Medical Research Institute (KEMRI), Nairobi, Kenya

**Keywords:** controlled cooking tests, clean cooking, fuel savings, Kenya, sub-Saharan Africa, energy efficiency

## Abstract

With a wide range of stoves and appliances available in the ever-evolving Kenyan cooking market, it is important to understand which options are the most cost, time and energy efficient to use. This information can help households to make more informed decisions about their energy use and policy makers to better understand which solutions to promote. Despite its importance, the existing literature offers scant evidence to guide optimal stove and fuel choices. In this research, we utilised controlled cooking tests to investigate the fuel required to cook six regularly prepared dishes on 10 prevalent stove and fuel combinations (including liquified petroleum gas, ethanol, charcoal, kerosene and electric appliances). We also tested the efficiency improvements from pre-soaking beans and using stovetop pressure cookers. We collected primary fuel cost data from across Nairobi in June 2023 and collated historical fuel prices from secondary sources spanning 2019–2023. The prices of liquified petroleum gas, charcoal and kerosene varied considerably by variables such as brand and location, whereas ethanol and on-grid electricity were more stable. The electric pressure cooker was the most cost- and energy-efficient device. For liquified petroleum gas and charcoal, combining pre-soaking beans with a pressure cooker substantially reduced fuel consumption, but was still costlier than the electric pressure cooker. The longitudinal comparison highlighted the dynamic nature of fuel prices in Kenya and how a household’s cost-optimal cooking stack can change at short notice. These findings demonstrate how comparative affordability varies both temporally and spatially and can be heavily affected by wider market and policy incentives.

## Introduction

Kenya is undergoing a demographic transformation that is characterised by a burgeoning middle class being subsumed into its metropolitan areas, causing urbanisation to increase at roughly 4% per year [1,2]. This presents challenges about how best to serve these growing cities with clean household energy, as required by Sustainable Development Goal 7 (SDG7), which calls for universal access to clean, affordable, modern energy by 2030 [3]. Electrification levels in Kenya’s cities are now high, but access to clean cooking is still lacking, with less than half of urban residents cooking primarily with clean fuels as of 2019 [4]. The Kenyan government has committed to meeting the SDG7 targets [5], with a national clean cooking strategy currently under development. This will involve the promotion of multiple fuels and technologies as illustrated by the formulation of an electricity-specific cooking strategy and policies for scaling up liquified petroleum gas (LPG) [6,7].

Kenya stands out as the sub-Saharan African country with the most advanced and diverse clean cooking sector. It has been the pilot market of choice for many of the most innovative clean cooking companies in the world (e.g., BURN Manufacturing, PAYGO Energy, Circle Gas and KOKO Networks), has its own clean cooking membership association (https://ccak.or.ke/) and is the epicentre of clean cooking research on the continent [8]. This has resulted in a wide range of clean and improved cooking solutions available to consumers alongside the traditional ones, each with its own unique set of advantages and disadvantages. It is therefore unsurprising that people tend to cook with multiple stoves and fuels, a practice known as ‘fuel stacking’ [9].

A literature review of fuel stacking revealed the complex decision-making that drives selection of stove and fuel for a given task, such as taste preferences, how much time is available and how many dishes are being prepared simultaneously [10]. However, it also found that most stacking was attributable to the environmental context and resources, and more specifically, to the affordability of different options. Clean cooking affordability is multifaceted, encompassing the stove cost, minimum fuel transaction size and the ongoing fuel cost [11–15]. Arguably the most important component is the latter aspect as it has the greatest impact on day-to-day decision-making. There is, therefore, a need to understand the comparative affordability of different cooking fuel and stove options in Kenya, in order to better inform consumers, policy makers and private sector players about the most cost- and energy-efficient technologies available.

The standard approach to understanding comparative affordability is through controlled cooking tests (CCTs), which examine the performance of different stoves in standardised cooking tasks. These tasks should consist of preparing a range of common dishes to reflect ‘the actual cooking that local people do every day’ ([16]: 1). The tests are typically performed by local people who are familiar with both the dishes and stoves of interest, and follow a standardised recipe designed to eliminate variability in the preparation method. CCTs thus facilitate the collection of locally relevant results to a reasonable degree of precision.

CCTs have been widely used across a number of different contexts. Most recently, the Modern Energy Cooking Services (MECS) programme has conducted CCTs in Zambia and Ethiopia to investigate potential energy, time and cost savings from using modern efficient cooking devices, and paired them with qualitative results to capture the eating quality of dishes [17,18]. Elsewhere, CCTs have been used to compare cooking with different basic and improved wood fuel stoves in Mexico, Tanzania and Haiti, for example, [19–21]. The comparison has been extended to include at least one modern energy cooking device in Benin and Ethiopia [22–23]. To the best of our knowledge, there is no published data on CCTs in Kenya, although the MECS eCooking Diaries collected detailed data on cooking energy, applicant use and food preparation over a 6-week period, thus providing rich but unstandardised data about real cooking [24]. A notable limitation of the studies to date is that they calculate the cost of preparing different dishes by assuming a single fuel price, usually the one paid by the research team at the time of the tests. In reality, fuel price varies with time, brand and place, a dynamic that is yet to be captured in a CCT analysis.

CCTs can also be used to address another gap in the academic literature, which concerns the most energy and cost-efficient way to cook long-boiling foods. Multiple research studies in East Africa have found that fuel stacking of charcoal alongside LPG is often driven by preferences to prepare long-boiling foods (e.g., beans and a Kenyan maize and bean stew known as githeri) on charcoal or firewood as it is cheaper [24–26]. Suggestions for addressing this include distributing stovetop gas pressure cookers, pre-soaking beans and co-provision of electric pressure cookers (EPCs) alongside LPG [27–30]. However, the comparative effectiveness of these solutions is yet to be satisfactorily investigated. Quantifying the financial, time and energy savings of preparing long-boiling foods could lead to insights about how to pair different fuels and efficient cooking practices together in order to drive more complete adoption of clean cooking.

Our study aims to address current gaps in the academic literature through a series of CCTs in Nairobi, Kenya with commonly cooked local dishes and fuels. The objectives of the study are to collect primary fuel price data from across Nairobi to estimate the most cost-effective way to cook; to identify the most energy and time-efficient cooking stack; to investigate how pressure cookers and bean soaking affect the energy and financial costs of cooking githeri and beans on different fuels; and to collate longitudinal fuel price data to examine how this has changed over time.

## Methods

### Fuel prices

The included fuels were decided based on the authors’ knowledge of the Kenyan market and were charcoal, LPG, ethanol, electricity and kerosene. Data was collected from around the Nairobi metropolitan area and included both ‘formal’ and ‘informal’ vendors and included data on price, brand and sales quantity. The formal channels consist of petrol stations and supermarkets that distribute major brands with fixed operating structures and margins. The informal retailers are usually smaller scale without official distribution licences. They tend to have lower operating expenses and are known for selling LPG cylinders that are not fully filled. We used a convenience sampling approach due to resource constraints, asking fieldworkers to take measurements from the areas in which they lived in order to get a spread throughout the city.

We weighed charcoal buckets and sacks for accuracy (taking care to exclude the containers, which remain with the vendor). We did not collect primary data about electricity prices as the majority of households in Nairobi use grid electricity, which has a fixed unit price set by the Kenyan Power and Lighting Company (KPLC).

The main round of data collection took place in the last week of June 2023, but under the new Finance Bill enacted in July, the government changed the value-added tax on LPG from 8% to zero-rated [31]. We therefore repeated the LPG price data collection at the beginning of August to examine whether the benefits of the tax change were passed onto end-users.

### CCTs

#### Stoves and dishes

**Figure 1:**
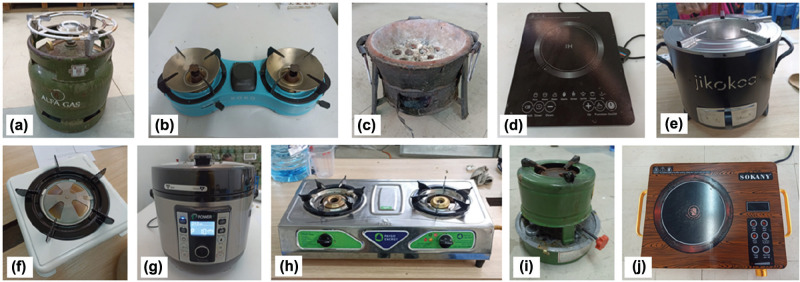
Stoves included in the CCTs, consisting of (a) single-burner screw top LPG (known locally as a meko); (b) liquid ethanol (KOKO); (c) charcoal jiko; (d) induction electric stove; (e) ICS (Jikokoa); (f) ethanol with gel fuel (Moto Sawa); (g) EPC; (h) two-burner LPG; (i) kerosene; and (j) infrared electric stove.

We followed Bailis’ CCT protocols in designing and conducting the tests [16]. The included stoves are shown in Fig. 1 a single-burner cylinder-top LPG stove known locally as a meko and referred to here as LPG-1, a basic kerosene stove, a two-burner liquid ethanol stove manufactured by KOKO Networks (Nairobi, Kenya), consisted of a basic charcoal stove known locally as a jiko, an improved charcoal stove (BURN Jikokoa, Nairobi, Kenya), an electric induction cooker (manufactured by IH), a single-burner gel ethanol cookstove produced by Moto Sawa (Nairobi, Kenya), an EPC (manufactured by PowerUp, Kampala, Uganda),^1^ a two-burner LPG stove manufactured by Real Flame (Haridwar, India) and connected to the cylinder by a regulator and a stove referred to here as LPG-2, an infrared electric cooker (manufactured by Sokany).

**Figure 2:**
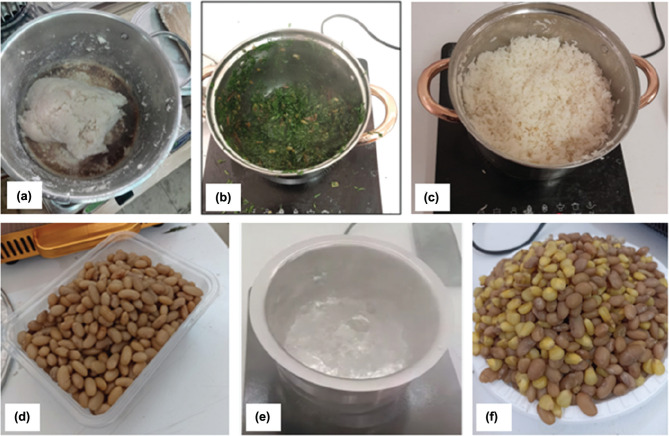
Dishes included in the CCTs consisting of (a) ugali; (b) sukuma wiki; (c) rice; (d) beans; (e) boiled water; and (f) githeri.

The included dishes are shown in Fig. 2 and were beans, githeri (a maize and bean stew), boiled water, sukuma wiki (a leafy green stir-fried vegetable), ugali (a dense porridge-like food made from maize flour) and boiled rice. The recipes are shown in Table A1 of the Appendix. These were selected because they are commonly prepared foods in Kenya that encompass a range of different cooking processes and durations (quick boiling, long boiling, frying and rigorous stirring).

We developed detailed procedures for cooking each food and aimed to cook four portions in the tests to mirror the average urban Kenyan household size [32]. The tests were performed by four local fieldworkers who were familiar with both the foods and stoves being tested. The full procedures incorporated the following considerations to standardise the tests as much as possible:

1. Specified the exact weight of ingredients, including water if there was a boiling step, and how they should be chopped.
2. Relied on observable rather than time-based factors to specify when to move onto the next step of the recipe. However, this was not possible for the tests involving electric and stovetop pressure cookers as the pot is sealed for the duration of cooking. In these cases, we conducted experiments prior to the tests to figure out the correct timing for preparation.
3. Specified the flame regulation for LPG, ethanol and kerosene stoves at each step in the protocol.
4. Specified the duration of soaking for beans and githeri, where applicable.
5. Developed a standardised procedure for lighting the charcoal stove.
6. Used standardised pots throughout the tests: a 28 cm aluminium sufuria, which our team believed to be the most commonly used pot in Nairobi households. However, aluminium pots are incompatible with induction stoves, so for these tests we used an identically sizes stainless steel pot.

Three repeats were conducted for each test, as recommended in Bailis’s CCT protocols [16]. Costs were calculated by multiplying the mean mass of fuel used in the test by the average fuel price per kilogram or per kWh (for electricity only). The price of ethanol was converted from litres to kg by dividing by its density (assumed to be 0.785 kg/L). The energy input was directly measured for the electrical appliance tests but was calculated using textbook specific energy values for the other fuels (LPG – 46 MJ/kg; charcoal – 30 MJ/kg; kerosene – 42.80 MJ/kg; ethanol – 27 MJ/kg. Source: https://www.engineeringtoolbox.com).

#### Effects of pre-soaking and LPG stovetop pressure cookers

As well as testing combinations of the included dishes and stoves, for beans and githeri we also tested the impacts of pre-soaking ingredients for 15 h and using a stovetop pressure cooker. To capture the sequential efficiency gains of cooking with charcoal, we also conducted CCTs where ugali and sukuma wiki were prepared consecutively. The data collected consisted of measuring the fuel used, which for charcoal, LPG and kerosene consisted of weighing the fuel before and after the tests, whereas for the electric cooking appliances we used using a standard plug-in electricity meter (resolution to 0.001 kWh), and measuring the duration of the tests.

## Results

### Fuel prices

**Figure 3:**
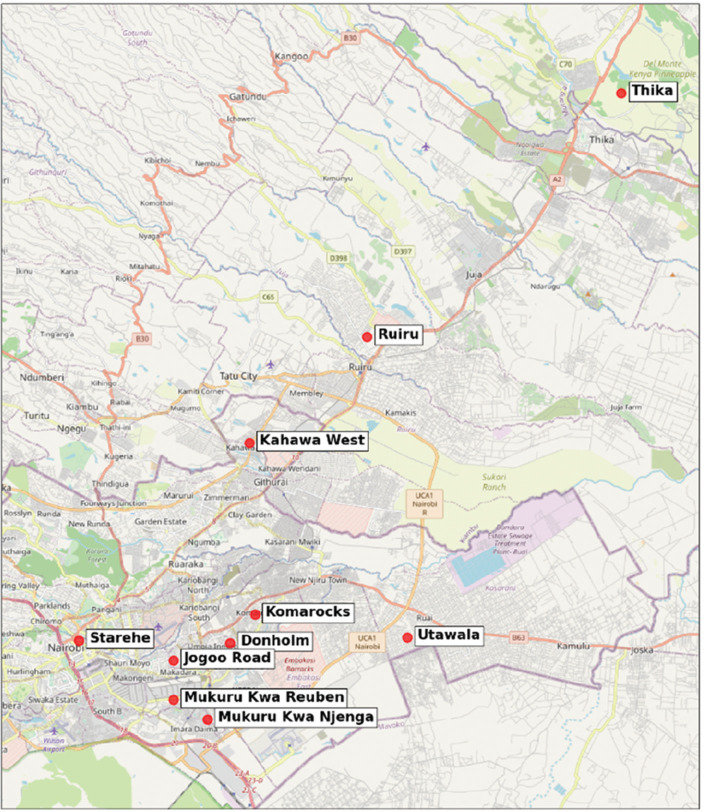
Map of fuel price data collection sites.

Fuel price data were collected in 10 locations around the Nairobi metropolitan area, as shown in Fig. 3. It encompassed two different ethanol technologies (KOKO, who supply a liquid ethanol fuel intended as a domestic cooking fuel, and Moto Sawa, who provide an ethanol gel intended as a camping fuel) and 10 LPG brands (Afrigas, Hashi, Jatel, KGas, Lake Gas, Oil Libya, ProGas, Sea Gas, SupaGas, Total). The full results are shown in the Appendix Table A2. The main electricity source in Nairobi is the electrical grid, which supplies power at a price fixed by the regulator, Kenya Lighting and Power Company (33 KSH/kWh at the time of data collection). Therefore, electricity prices are not discussed further in this section.

#### Brand variation

The cost per kg of LPG varied between brands by a maximum of 24%, with KGas having the minimum average brand price (192 KSH/kg) and AfriGas the maximum (253 KSH/kg). Brand pricing also fluctuated between retail points; for example, KGas cost 50% more in Utawala (231 KSH/kg) than in Mukuru Kwa Njenga (154 KSH/kg).

There was substantially greater variation between ethanol brands and technologies, with Moto Sawa averaging more than three times the price of KOKO (291 KSH/L vs. 85 KSH/L). However, we found minimal variation between retail points (KOKO has a fixed price nationally and we could only find Moto Sawa in one supermarket).

#### Sale quantity variation

Kerosene was always sold in 1 L bottles. Charcoal was sold in buckets (roughly 5 kg) or tins (roughly 1.5 kg) and was cheaper when purchased in smaller quantities (average of 77 KSH/kg for smaller tins vs. 85 KSH/kg for larger buckets). A similar trend held for LPG, which was sold in 13 kg and 6 kg bottles, with the smaller bottle being slightly better value (average of 216 KSH/kg for 13 kg cylinders and 213 KSH/kg for 6 kg bottles). KOKO ethanol is dispensed from kiosks at a fixed price per litre set centrally by the company, so is independent of purchase quantity. Moto Sawa ethanol was sold in 3 L or 0.5 L bottles with a slightly cheaper rate for the larger bottle (274 KSH/L for 3 L vs. 308 KSH/L for 0.5 L).

#### Location variation

**Figure 4:**
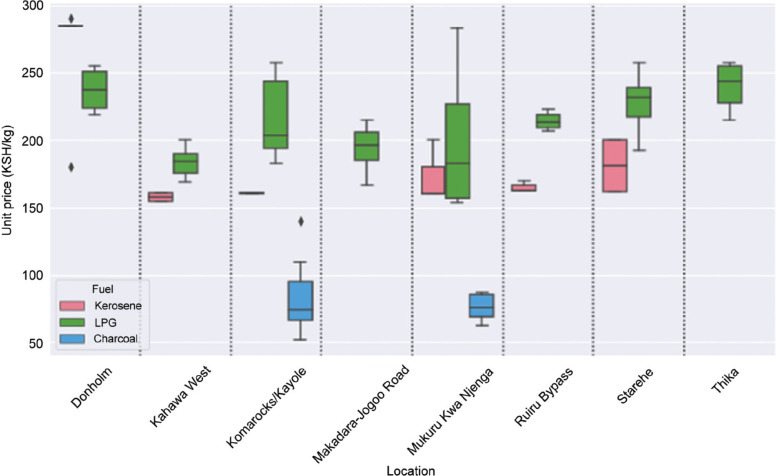
Fuel price variation by location.

Figure 4 visualises the price variations in different locations throughout Nairobi, limited to LPG, kerosene and charcoal as these were the fuels with the most variation and data points. The graph shows that whilst the ranking of fuels was mostly consistent (LPG > kerosene > charcoal), there was considerable variation between locations. For example, the mean price of LPG in Thika (240 KSH/kg) was 30% higher than in Kahawa West (184 KSH/kg).

#### Vendor type variation

Data for kerosene and LPG prices were collected from petrol stations – that is, licensed, regulated retail points – and informal, unregulated street sellers. The mean price of kerosene differed marginally between these two locations (184 KSH/L from street vendors and 182 KSH/L from petrol stations, a difference of 0.6%), but LPG was cheaper on the street (204 KSH/kg for street vendors vs. 226 KSH/kg for petrol station vendors, a difference of 11%).

#### LPG prices before and after the VAT policy change

LPG price data was collected in June 2023, when the VAT rate was 8%, and in August 2023, when the VAT rate was cut to 0%. LPG was 15% cheaper in August, with Table 1 showing there were similar reductions observed at both petrol station and street retail points, and a larger price fall on 6 kg bottles than 13 kg bottles (−17% vs. −12%, respectively).

**Table 1.** LPG prices before (June 2023) and after (August 2023) the introduction of the 2023 Finance Bill, which reduced VAT on LPG from 8% to 0%

Sales quantity/location	August 2023	June 2023	Change
	Mean price (KSH/kg)	Mean price (KSH/kg)	
13 kg	190	216	−12%
6 kg	177	213	−17%
Petrol station	194	226	−14%
Street	172	204	−15%

### CCTs

#### Stove and dishes

**Figure A1:**
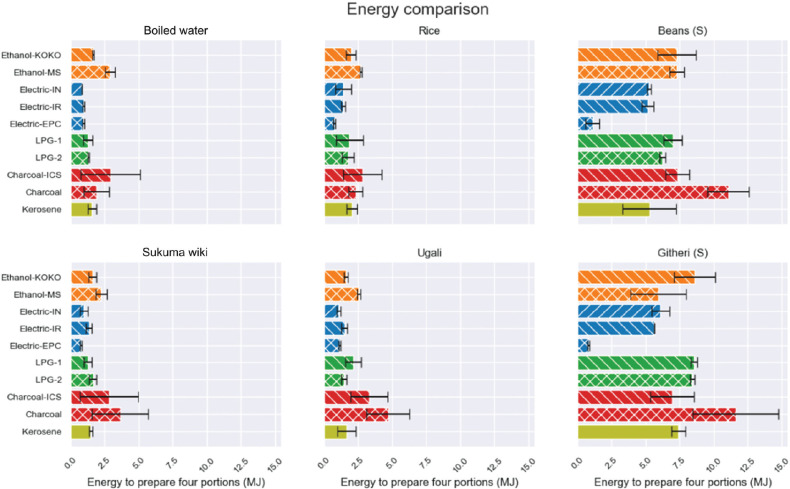
Energy to cook four portions of food. Ethanol – KOKO = KOKO liquid ethanol stove; ethanol – MS = Moto Sawa gel ethanol stove; electric – IN = electric induction stove; electric – IR = electric infrared stove; electric – EPC = electric pressure cooker; LPG-2 = two-burner LPG stove; LPG-1 = screw top one-burner LPG meko stove; charcoal – ICS = improved charcoal stove; charcoal = standard charcoal jiko; kerosene = standard kerosene stove. Each test was repeated three times and the black bars show the standard errors.

**Figure A2:**
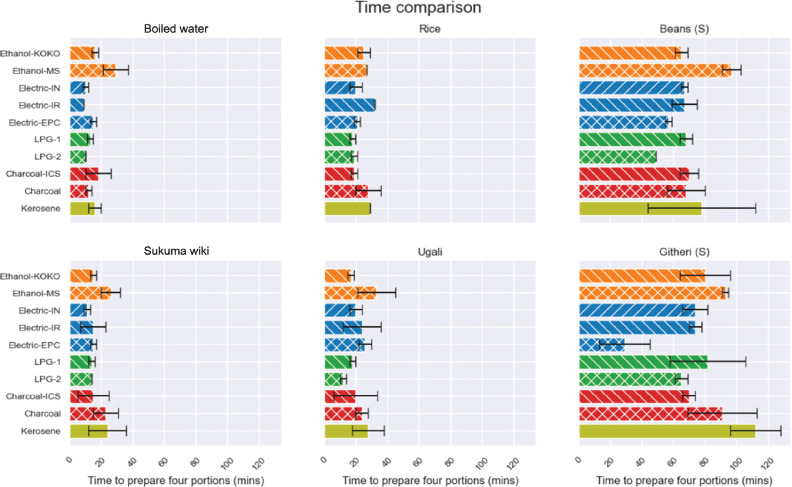
Time to cook four portions of food. Ethanol – KOKO = KOKO liquid ethanol stove; ethanol – MS = Moto Sawa gel ethanol stove; electric – IN = electric induction stove; electric – IR = electric infrared stove; electric – EPC = electric pressure cooker; LPG-2 = two-burner LPG stove; LPG-1 = screw top one-burner LPG meko stove; charcoal – ICS = improved charcoal stove; charcoal = standard charcoal jiko; kerosene = standard kerosene stove. Each test was repeated three times, and the black bars show the standard errors.

The full results of the CCTs are shown in Table A3 of the Appendix. The findings presented below focus primarily on the cost of cooking, but the energy and time results are also described in the text and corresponding figures visualising their results are included in Figs. A1 and A2 of the Appendix. Table 2 shows the prices assumed for each fuel, which were calculated from the mean of the fuel price results collected in July (when LPG was still taxed at 8%).

**Table 2.** Fuel prices used in the CCT cost calculations

	Mean price (KSH)	Unit	Source
LPG	214	KSH/kg	Primary data collection June 2023
Charcoal – ICS	79	KSH/kg	Primary data collection June 2023
Charcoal	79	KSH/kg	Primary data collection June 2023
Electricity	33	KSH/kWh	KPLC tariff
Kerosene	183	KSH/L	Primary data collection June 2023
Ethanol – KOKO	85	KSH/L	Primary data collection June 2023
Ethanol – Moto Sawa	291	KSH/L	Primary data collection June 2023

#### Dish-level fuel comparison

**Figure 5:**
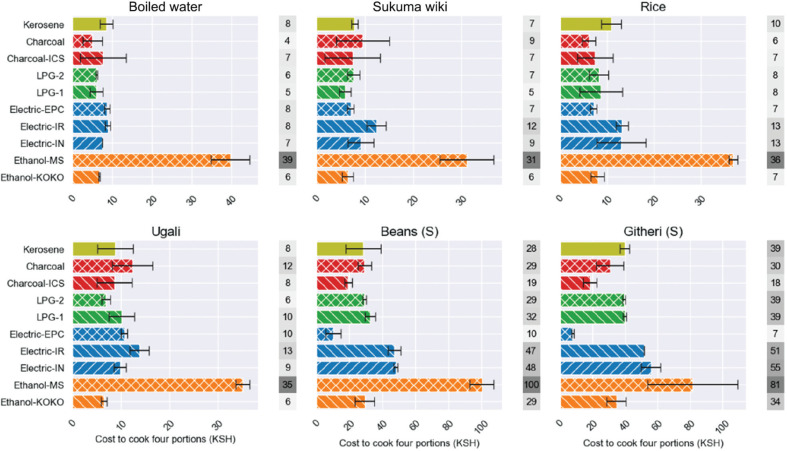
Cost to cook four portions of food. Ethanol – KOKO = KOKO liquid ethanol stove; ethanol – MS = Moto Sawa gel ethanol stove; electric – IN = electric induction stove; electric – IR = electric infrared stove; electric – EPC = electric pressure cooker; LPG-2 = two-burner LPG stove; LPG-1 = screw top one-burner LPG meko stove; charcoal – ICS = improved charcoal stove; charcoal = standard charcoal jiko; kerosene = standard kerosene stove. Each test was repeated three times, and the black bars show the standard errors.

Figure 5 compares the cost of cooking each of the six CCT dishes across the 10 stoves. The most expensive fuel/stove combination to use was Moto Sawa ethanol, typically costing twice as much as the next most expensive option for each of the tests and averaging at three times the price of KOKO ethanol.

The EPC was the most cost-effective electric cooking appliance and the cheapest mode of cooking overall. This was driven by its excellent performance for preparing long-boiling foods (githeri and beans); it was comparable to LPG and charcoal across the other four dishes. The induction and infrared stoves performed similarly and tended to be the second most expensive options after Moto Sawa ethanol.

LPG-2 was expected to be more efficient than LPG-1. This is because the two-burner stove (LPG-2) was connected to the cylinder via a pressure regulator, whereas the screw top single-burner (LPG-1) was directly connected to the cylinder valve, causing a less controlled release of gas. However, we found that LPG-1 and LPG-2 yielded similar test results, with these stoves being consistently cheaper than the induction, infrared and Moto Sawa ethanol stoves. They performed similarly to the two charcoal, EPC and kerosene stoves for boiled water, sukuma wiki, rice and ugali. For the long-boiling foods, they were similar to KOKO ethanol and kerosene, but significantly more expensive than the charcoal stoves and EPC.

The improved charcoal stove (ICS) used 24% less charcoal than the jiko and therefore cost a quarter less to run. The ICS was more expensive than the jiko for the boiled water and rice tests, but there were particularly large standard errors associated with these tests, making their results less reliable.

Charcoal consumed the most energy across the tests (see Appendix Fig. A1). Ethanol, LPG and kerosene were very similar although kerosene used slightly less for long-boiling foods. The electric cooking devices were the most energy-efficient but exhibited different patterns for different dishes. The induction stove was generally more efficient than the infrared stove. The EPC was less comparatively efficient for boiling water, a short-boiling activity that is of insufficient length for the EPC’s pressurisation feature to be of benefit, and for preparing ugali, which requires constant stirring and therefore no use of a lid. However, the EPC significantly outperformed all other stoves for long-boiling foods, using five times less energy than the next closest option (electric infrared) and 10 times less energy than charcoal.

The time results are shown in the Appendix Fig. A2. Kerosene was the slowest stove overall, followed closely by charcoal, with the former averaging 40% slower than the fastest cookstove (EPC). The induction stove was the fastest electrical appliance for boiling water, sukuma wiki, rice and ugali. The EPC’s advantage primarily came from githeri, which it cooked in just half an hour, taking half the time as then next closest stove (LPG LP, 65 mins) and 3.5 times faster than kerosene (112 mins).

#### Optimal cooking stacks as of June 2023

**Figure 6:**
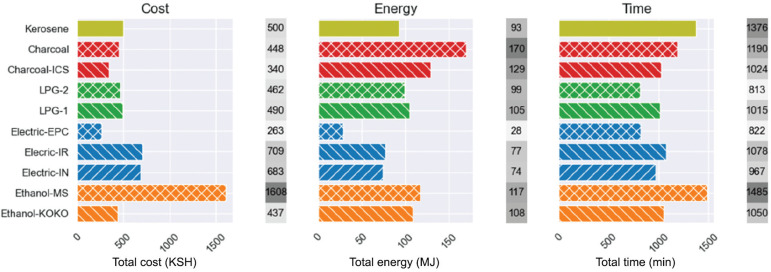
Estimated cost, energy and time to prepare one week of meals.

Leary [33] conducted a detailed study of household cooking practices in Nairobi and produced a template of the typical urban Kenyan diet. Based on this research, we assume that in an average week a Kenyan household might prepare sukuma wiki six times, beans five times, rice six times, githeri four times, boiled water five times and ugali five times. These figures were used to estimate the cost, energy and time required to cook with each stove for one week in June 2023, as shown in Fig. 6.

The graphs reflect the dish-level findings. In terms of cost, Moto Sawa ethanol is the most expensive stove to use (1608 KSH/week), followed by the electric induction and infrared stoves (684 and 710 KSH/week, respectively). KOKO ethanol, the two LPG stoves, kerosene and charcoal are similarly priced, ranging from 438 KSH/week (KOKO ethanol) to 500 KSH/week (kerosene). The EPC was the cheapest stove to use (264 KSH/week) and was 29% cheaper than the next most economic stove (ICS, 341 KSH/week) and six times cheaper than the Moto Sawa ethanol stove.

Moto Sawa ethanol performed notably better in the energy results (117 MJ/week), where it was not penalised for its high unit price, and yielded results similar to KOKO ethanol (109 MJ/week). Charcoal was the least efficient fuel, although the ICS (129 MJ/week) used a third less energy that the jiko (170 MJ/week). The LPG-1 and LPG-2 used a similar amount of energy to the kerosene stove and roughly a third more energy than the induction and infrared stoves (99, 105, 75 and 77 MJ/week, respectively). The EPC was also by far the most energy-efficient stove (29 MJ/week) and used around six times less energy than the charcoal jiko.

The time results were more homogenous, with the slowest stove (Moto Sawa ethanol, 25 hours/week minutes/week) taking less than twice the time to use as the fastest stove (one-burner LPG, 13.5 hours/week).

#### Optimal cooking stack scenarios

**Figure 7:**
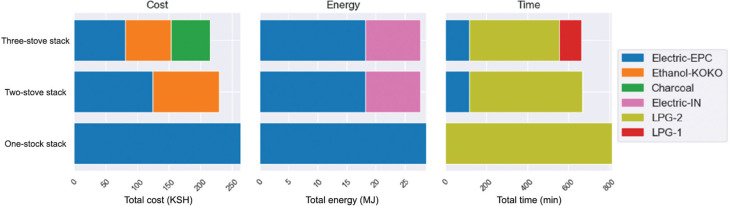
Optimised one-, two- and three-stove stacks for weekly cost of cooking, energy consumed, and time spent cooking.

The results of the dish-level CCTs also allowed us to calculate the optimal combinations of stoves to cook with for a typical Kenyan diet, as described in the previous section. This was achieved by considering the full range of possible stove combinations and assuming that, for each combination, the most efficient stove-dish pairs were used. The results are displayed in Fig. 7, and also segregate the contribution of each stove to the stack. The most cost-effective two-stove stack was EPC + KOKO ethanol (230 KSH/week) and three-stove stack was EPC + KOKO ethanol + charcoal (215 KSH/week). The gains compared to using just the EPC were 13% and 18%, respectively. The most energy-efficient two-stove stack was EPC + electric induction (29 MJ/week), but this was only a 3% improvement on using the EPC alone. There was no more efficient three-stove combination. The fastest cooking two-stove stack was the one-burner LPG meko + the EPC (11 h 9 min) and the three-stove stack also incorporated the two-burner LPG stove (11 h 3 min). However, the advantage of shifting from the two-stove to three-stove stack was marginal (<1% time saving).

#### Sequential charcoal comparison

The above sections assume that the test results are additional. However, this may not be the case for charcoal; unlike the other stoves included in the tests, the jiko and ICS cannot be instantly turned on and off as the flame takes time to be ready to cook and is often left to die down rather than being extinguished. This could cause the optimal cooking stack results to be biased against charcoal. To test this, we compared the fuel required to cook sukuma wiki and ugali (a common meal combination in Kenya) both sequentially (i.e., by preparing immediately one after the other) against the results generated from summing the individual dish tests. We found that almost an identical mass of charcoal was consumed in the tests (average of 43.2 g for non-sequential vs. 43.3 for sequential).

### Effects of pre-soaking and LPG pressure cookers

**Figure 8:**
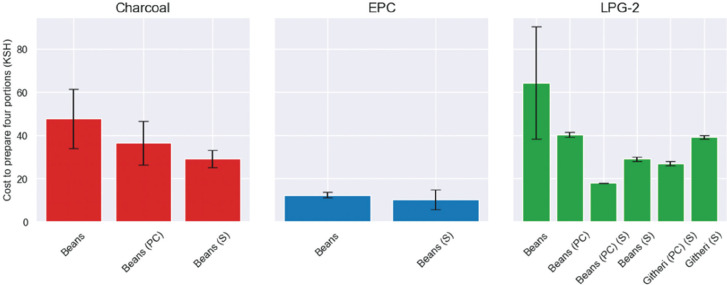
Comparing the cost of cooking hard foods on the charcoal jiko, EPC and one-burner LPG meko. Beans = unsoaked beans; beans (PC) = unsoaked beans cooked in pressure cooker; beans (S) = soaked beans; beans (PC) (S) = soaked beans cooking in pressure cooker; githeri (PC) (S) = soaked beans and maize cooked in pressure cooker; githeri (S) = soaked beans. Each test was repeated three times and the black bars show the standard errors.

Figure 8 shows the costs of cooking long-boiling foods on charcoal, EPC and the two-burner LPG stove with different combinations of soaking and use of stovetop pressure cookers. Soaking was more effective than using a pressure cooker: for beans prepared on both the charcoal and the LPG stoves, the pressure cooker reduced the cost of preparation (25% saving for charcoal and 38% for LPG), and soaking by twice as much (40% saving for charcoal and 55% for LPG). For LPG, combining soaking with the pressure cooker resulted in a three-fold cost saving compared to preparing unsoaked beans, but was still twice the price of using an EPC. The githeri results also showed the efficacy of combining the pressure cooker with soaking (31% improvement on soaking alone). Surprisingly, soaking the beans had negligible impact for the EPC.

### Temporal variation

**Figure 9:**
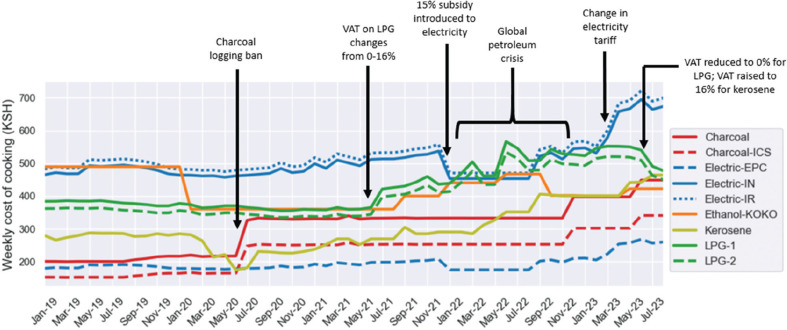
Temporal variation in the cost of weekly cooking. The annotations highlight some global events and policy changes that impacted the price of fuels. Note that there were substantially fewer price data points for KOKO ethanol and charcoal compared to the other fuel. Moto Sawa ethanol is excluded due to lack of data. Data sources: charcoal, kerosene and LPG – KNBS; electricity – Stimatracker; KOKO ethanol – provided by KOKO.

Figure 9 combines the CCT results with historic fuel price data to estimate how the cost of cooking a typical Kenyan menu for four people on a range of stoves and fuels varied from 2019 to 2023. The graph shows that the comparative cost of using different stoves is highly dynamic, with the ranking of fuels regularly changing and sensitive to both policy changes and global events. Since the enforcement of the charcoal logging ban in 2020, the EPC has been the cheapest cooking appliance overall. KOKO ethanol has had the most stable price throughout the time period considered. Induction cookers were cheaper than LPG until the beginning of 2023, when electricity tariffs were raised substantially to account for additional charges of power generation, the weak foreign exchange rate, increasing rates of inflation, a new rural electrification levy and 16% VAT introduction [34]. There has been a general upward trend in prices since 2021 (with the exception of ethanol and LPG), which could be explained by the weakening trajectory of the Kenyan shilling against the US dollar that started in the second half of 2021 and continues today.

## Discussion

This study presented the results of CCTs in Nairobi accompanied by extensive fuel price data collection around the city and collation of historical fuel price data. We measured the time and quantity of fuel required to cook six dishes across 10 different cookstoves and used the test results to calculate the financial and energy costs of preparing each dish and stove combination. We also examined the impacts of pressure cooker and soaking interventions on the preparation of long-boiling foods. Although the price results are unique to the snapshot of time and space in which our research took place, the fuel consumption results from the CCTs allow the analysis to be easily updated with new price data. These results are also likely to be generalisable to other urban East African contexts with relatively similar dietary patterns (e.g., Tanzania, Uganda and Rwanda, by inputting local fuel prices).

Ethanol and electricity prices were fixed by providers whereas prices for charcoal, kerosene and LPG varied spatially. This could be attributed to varying density of points of sale, localised demand and accessibility, as has been found for fuel prices elsewhere [35,36]; we have also heard unverified anecdotes about vendors in specific localities collaborating to artificially hike prices. It was slightly cheaper to buy kerosene and LPG from unregulated street vendors than petrol stations. The reason for this is unclear but could be related to under-filling of cylinders at unregulated sales points.

LPG and ethanol prices also varied by brand. LPG is an imported fuel with prices primarily affected by the landed commodity cost and margins for retailers, distributors and wholesalers [37]. Therefore, variations in company margins, which are in turn determined by their fixed costs and margin structures, are likely to explain the differences between brands. Ethanol is also mostly an imported fuel subject to similar forces, but these dynamics alone cannot explain the extremely large differences in the prices of KOKO and Moto Sawa ethanol. Other contributing factors could be economies of scale and use of carbon credits. KOKO’s technology is widely available in Kenya, where they have reached over 750,000 households [38], whereas Moto Sawa is not a mainstream cooking solution and could only be found in one supermarket during our data collection. There are additional engineering and distribution costs that could also contribute to the higher price for Moto Sawa, such as a more complex manufacturing process requiring the addition of a gelling agent and the need for durable single-use packaging, as opposed to KOKO’s reusable ethanol bottles.

KOKO also benefit from carbon credits, a tool playing an increasingly important role in driving affordability of innovative clean cooking solutions. In KOKO’s case, carbon credits subsidise both the stove and fuel, reportedly reducing KOKO’s fuel price by 25–40% [39]. If this is true, then our findings demonstrate the important role this subsidy currently plays in making ethanol a cost-competitive household fuel. Carbon credits offer the exciting potential to bring this climate-friendly, renewable cooking fuel to new populations. They also provide KOKO with buffer to stabilise their prices during periods of wholesale market volatility, as demonstrated by Fig. 9. However, credit integrity for clean cooking is facing increasing levels of scrutiny from academic researchers and rating agencies, who have criticised inflated assumptions relating to the baseline consumption of polluting fuels, the fraction of non-renewable biomass comprising baseline fuel use and the assumed usage of stoves once delivered [40–42]. These factors combine to increase the likelihood that developers are failing to contribute the climate benefits that they claim. This risk of over crediting, combined with high levels of price volatility within the carbon market [43], raises questions about the long-term ability of ethanol companies to subsidise fuel purchases.

Nevertheless, recent developments suggest a movement towards higher project quality through improvement in credit certification methodologies and active efforts from governments to tackle over crediting through more conservative default assumptions [44–46]. These changes suggest a shift towards lower numbers of credits being generated in the hope that these more credible credits can attract a higher price. The price per tonne of carbon avoided will be influenced by factors within the voluntary carbon market and bilateral trading arrangements enforced through Article 6 of the Paris Agreement. For example, the Kenya Climate Change Amendment Act adds significant clarity to how the Kenyan carbon market will be regulated to improve the integrity and fairness of carbon flows, boosting confidence of carbon project developers, investors and buyers [47].

The LPG prices collected in August were 15% cheaper than those collected in June, showing that the 8% VAT scrap could not be the only factor driving the decrease in price. The same patterns of price reduction were also widely reported by the Kenyan media [48,49]. Yet they contradicted trends elsewhere, as the average global propane price *increased* by 13% over the same period [50]. Kenya’s exception could be due to the concurrent removal of Kenya’s railway development levy at 2.5% [31] and steady decreases in propane prices from a specific LPG provider, Saudi Aramco (Dhahran, Saudi Arabia), who hold large contracts with the Government of Kenya [51,52].

The EPC was the most cost-efficient cooking appliance as it vastly outperformed other stoves for preparing long-boiling foods, corroborating the findings of multiple other studies that advocate the advantages of EPCs [24,53,54]. This is because pressure cookers work by trapping steam inside the cooking vessel, which increases the temperature and pressure so that food cooks faster and more evenly. The fast-cooking dishes which were fried did not make use of the pressurised feature, explaining why the EPC exhibited such large advantages only on long boiling. Small savings of 13% could be yielded by stacking it alongside KOKO ethanol. The EPC was followed closely by the two charcoal stoves and then the KOKO ethanol stove. The next cheapest modes of cooking were the kerosene and LPG stoves, which performed similarly. The most expensive (by a considerable margin) was the Moto Sawa ethanol stove.

The BURN Jikokoa improved cookstove used 24% less fuel than the charcoal jiko – less than the 39% fuel savings revealed by an independent study of 1000 households adopting the stove in Nairobi [55]. Although Berkouwer’s study benefits from a large sample size and the study of real-world households, charcoal usage data was calculated based on self-reported purchasing patterns and weighing of ash rather than by using pre-post cooking event weight measurements. This illustrates the difficulty of accurately measuring household-level fuel use and determining the savings associated with adopting a particular stove – it is simply impractical to weigh fuel before and after each cooking event for large numbers of participants. Therefore, our figure, which is based on precise and comparable measurements, is likely to be more accurate.

The electric cooking appliances were the most energy efficient with the EPC again taking the lead. The most energy-efficient cooking stack was the EPC combined with the induction stove, although the gain was marginal (3%) compared to the EPC alone. The two charcoal stoves were the least energy-efficient devices tested, although the ICS did use a quarter less energy than the standard jiko. However, our results are likely biased towards the electrical stoves as we did not consider the energy conversion involved in the electricity generation step. Full life cycle analyses are required to accurately compare between fuels.

We found clean stoves and fuels tended to be quicker to cook with than their polluting counterparts. The quickest stoves overall were the one-burner LPG stove and the EPC, but this does not necessarily hold if a household were to solely adopt these stoves. This is because they are both one-burner devices that cannot heat multiple pots simultaneously – a limitation found to drive fuel stacking across multiple contexts [56–58]. In practice, the most convenient modes of cooking are likely to be two-burner clean cookstoves, such as KOKO ethanol or a double-burner LPG stove.

Beans and githeri are long-boiling foods that have been widely found to perpetuate the use of charcoal [24–26]. Indeed, it was cheaper to prepare these foods on charcoal than on LPG, although the gap diminished when the beans and maize were pre-soaked or prepared in a pressure cooker. Our findings showed that soaking alone was more effective than using a pressure cooker alone, but that combining these interventions yielded substantial further fuel savings. Initiatives promoting pressure cookers as fuel-saving devices, such as the pilot in Cox’s Bazaar displacement camp in Bangladesh [30], should therefore also encourage recipients to soak pulses prior to cooking. Further work is required to understand the cultural compatibility of these practices. Importantly, even with pre-soaking and use of stovetop pressure cookers, the EPC vastly outperformed the LPG and charcoal stoves in terms of cost and energy consumption.

It is important to highlight that the input data for this cost comparison came from fuel price data collected in June. If recalculated using the August data then LPG would have been cheaper than kerosene and comparable to KOKO ethanol, demonstrating the dynamic hierarchy between fuels. This is amplified by the spatial variation in some fuel prices throughout the city, meaning that the comparative costs are also neighbourhood dependent. Having access to a range of cooking technologies allows Kenyan consumers to respond to such price changes by altering their fuel stack. Such behaviour is a livelihoods strategy that has been observed in many cases; for example, hikes in LPG prices in 2022 resulted in news reports across six countries about households reverting to cooking with charcoal [59], and the removal of LPG subsidies in Ghana was similarly associated with increases in biomass fuel consumption [60]. These findings reinforce the importance of consumers having access to multiple clean cooking options, which allows them to continue accessing clean energy even if one fuel surges in price.

The transient relevance of the cost of cooking at a single point in time is further demonstrated by the longitudinal comparison between different stoves and fuels shown in Fig. 9. Cooking prices in Kenya are closely intertwined with both the local and global economy, resulting in an ever-changing optimum fuel stack; for example, there are times when it is financially advantageous for a household to switch from mostly relying on their charcoal jiko to their LPG meko, and others where substantial cost savings can be realised through eliminating LPG altogether. This may help explain why even Kenyan households with relatively few material possessions often own so many different types of stoves. Such a strategy allows them to amend their cooking practices in response to fuel price changes, fuel shortages and equipment breakages. Generating clear, understandable consumer price indexes for cooking fuels could allow households to make even more informed decisions about how they cook, and which stoves they should invest in next.

Finally, our findings point towards a number of important factors that impact the affordability of different cooking fuels and therefore their use. Some are outside the control of stakeholders driving clean cooking transitions, such as currency exchange rates; global commodity prices, including carbon credits; and health of the economy, which affects capacity to pay. Others are not, such as the technical details of carbon credit methodologies, which affect the yields claimed by providers; taxation and subsidy regimes for different fuels; and policies intended to directly impact fuel supply, such as logging bans. To accelerate the uptake of clean cooking fuels, as required by SDG7, these different levers must align to create disincentives for use of polluting fuels and incentives for adoption of clean ones. For example, the 2023 raises in electricity tariffs contradict the Kenyan government’s aspirations to widen adoption of electric cooking, and Kenya’s 30% import tax on ethanol fuel limits its penetration to lower-income households.

### Limitations

In reality, each household’s cooking practices are unique, using their own recipes, preparing different quantities of food, and interacting with the stove in different ways. As Scott explains, the CCT approach ‘takes the test one step further away from the real-world experience that we want to understand’ [61]. This means that our results are useful for comparing across different fuels but do not represent the lived experiences of real-world households.

Despite our best efforts, the standard error for many of the results was still high (median coefficient of variance of 9% with a range from 0% to 38%), especially for the charcoal tests, which were harder to control (median 14%). We therefore agree with Wang et al.’s conclusion that three repeats are not always adequate for CCTs, especially for charcoal stoves, and suggest that Bailis’ official CCT protocol should be updated to reflect this [16,62]. The fuel price data was collected via convenience sampling because we did not realise in advance of the study how wide the variation would be. This means that the numbers we obtained are likely to be biased and not representative of Nairobi as a whole. These limitations mean that this study should be treated as a pilot; we recommend that future studies use a more systematic approach to fuel price data collection and conduct a larger number of repeats in order to obtain more reliable data.

Our study had a focus on understanding the relative affordability of different cooking fuels. However, affordability has multiple other components that have not been included in our analysis. For example, the EPC was the cheapest appliance to run but had the highest appliance cost at 12,000 KSH – 27× the price of the charcoal stove. The minimum recorded purchase of charcoal (30 KSH for a tin) was similarly an important advantage over LPG and kerosene, which respectively had minimum purchase quantities of 158 KSH (6 kg cylinder) and 155 KSH (1 L). These financial barriers must also be considered to enable Kenyan consumers to transition to optimal cost- and energy-efficient cooking stacks.

## Conclusions

With a wide range of stoves and appliances available in the ever-evolving Kenyan cooking market, it is important to understand how fuel prices vary throughout the city, over time and which options are the most cost, time and energy efficient to use. Extensive fuel price data collection showed that prices of LPG, ethanol, kerosene and charcoal varied throughout the city primarily by brand, location and vendor type. Fuel prices also varied widely and frequently over time, showing that a household’s cost-optimal fuel stack is not fixed. We also found that the price of LPG fell drastically from June to August 2023, coinciding with (but not solely due to) government tax policy changes, and showed how carbon credits increase the competitiveness of ethanol cooking. These findings demonstrate how comparative affordability varies both temporally and spatially and can be heavily affected by wider market and policy incentives.

The EPC was the most cost- and energy-optimal stove by a substantial margin, even outperforming pre-soaked pulses prepared in stovetop pressure cookers on charcoal and LPG. It was also convenient, taking a similar amount of time to prepare a typical Kenyan menu as LPG, which is often flagged as the fastest way to cook. However, in reality EPCs are limited by their high price and by only being able to prepare a single dish at a time. Developing two-pot EPCs and bespoke financing mechanisms could allow households to fully capitalise upon the benefits of this exceptionally efficient device.

## Data Availability

Data sharing not applicable to this article as no datasets were generated or analysed during the current study.

## Note

1 EPCs are heated by a resistive heat plate embedded in their base. They consist of a sealed chamber that traps steam as its contents are heated, thus building pressure and driving the boiling point of water past 100 °C. This accelerates the boiling process, allowing the contents of the pot to cook faster and more efficiently.

## Appendix

**Table A1.** Recipes for CCTs

Test	Ingredients	Instructions
Boiling water	2000 mL of water	Heat the water in a sufuria. Turn off as soon as it gets to a full rolling boil (when the bubbles are rapidly breaking the surface).
Rice	500 g rice 1200 mL of water 1 teaspoon salt 1 tablespoon oil	Bring the water to boil in a medium saucepan. Add the salt and oil. When the water has returned to a boil, stir in the rice. Let the water return to a light simmer. Stir again, cover the pot and turn the heat down to low. Keep the rice simmering slightly, and keep the pot covered. Once all the water is absorbed, taste by trying a grain for tenderness – further there should be the appearance of small holes on the surface and all the water is absorbed.
Plain boiled beans	800 g beans 1600 mL water Salt	Soak the dried beans in water overnight for 8 h. Drain the soaked beans and rinse them thoroughly. Put the beans in a pot and add water. Bring to a boil over high heat. Once boiling, reduce the heat to medium and simmer. Simmer the mixture until the beans are tender. This may take 1.5–2 h for dried ingredients (less if you use a pressure cooker). Keep an eye on the pot and add more water as needed to prevent drying out. Taste with some beans on your tongue and mash them towards the roof of your mouth. This should feel effortless, with no traces of firmness.
Ugali	500 g maize meal/corn meal 1100 mL water	Heat water over medium heat in a deep pan. Make sure you use a pan with a handle. Sprinkle about 1 tablespoon of the maize meal while the water comes to a boil. Add the maize meal or cornmeal and keep on stirring with a strong wooden spoon. Keep stirring and pressing the mixture against the sides of the pan to break up the lumps. As the mixture becomes thicker, it becomes more difficult to mix but keep mixing and breaking up the lumps. Cook until the mixture begins to come away from the sides of the pan.
Sukuma wiki	600 g of sukuma wiki (collard greens) leaves 170 g red tomatoes 20 g onion ¼ cup oil	Wash the collard greens then use a knife to chop off the stems. Discard the stems (you only require the leaves). Wash and chop the tomatoes, peel and slice the onion. Next, heat your cooking pot on the stovetop and set it to heat under medium heat. Next, add in the cooking oil and allow it to heat for about 1–2 min. Fry the onion until golden brown. Next, add the tomatoes and allow them to cook. The tomatoes should be totally soft. Once the tomatoes are totally cooked through, add the sukuma wiki leaves and some salt. Cover and allow it to reduce for about one minute then stir. Cook for a further until all the water has dried up.
Plain Githeri	700 g dried beans 300 g fresh maize 2000 mL Water	Soak the dried beans in water overnight for 8 h. Since maize is fresh, soaking is unnecessary. Drain the soaked beans and rinse them thoroughly. In a pot, combine the maize and beans. Add water. Bring to a boil over high heat. Once boiling, reduce the heat to medium and simmer. Simmer the mixture until both the maize and beans are tender. This may take 1.5–2 h for dried ingredients (less if you use a pressure cooker). Keep an eye on the pot and add more water as needed to prevent drying out. Taste with a combination of beans and maize on your tongue and mash them towards the roof of your mouth. This should feel effortless, with no traces of firmness.

**Table A2.** Fuel prices in Nairobi, June 2023

Brand	Sales qty	Location	Vendor type	Fuel	Min price/unit	Mean price/unit	Max price/unit	Number of data points
Brand variation								
Afrigas	—	–	–	LPG	250	254	255	4
Hashi	–	–	–	LPG	200	200	200	1
Jatel	–	–	–	LPG	217	217	217	1
KGas	–	–	–	LPG	154	192	231	17
Lake Gas	–	–	–	LPG	219	219	219	1
Oil Libya	–	–	–	LPG	223	228	233	2
ProGas	–	–	–	LPG	167	207	233	7
Sea Gas	–	–	–	LPG	225	225	225	1
SupaGas	–	–	–	LPG	210	210	210	1
Total	–	–	–	LPG	183	237	283	13
KOKO	–	–	–	Ethanol	78	85	95	10
Moto Sawa fuel gel	–	–	–	Ethanol	274	291	308	2
Sales quantity variation								
-	Bucket (~5 kg)	–	–	Charcoal	67	85	110	6
-	Tin (~1.4 kg)	–	–	Charcoal	52	77	140	14
KOKO	–	–	–	Ethanol	78	85	95	10
Moto Sawa	3 l	–	–	Ethanol	274	274	274	1
Moto Sawa	0.5 l	–	–	Ethanol	308	308	308	1
All	13 kg	–	–	LPG	154	216	256	23
All	6 kg	–	–	LPG	158	213	283	27
Location variation								
-	–	Mukuru Kwa Njenga	–	Charcoal	62	77	87	11
-	–	Komarock/kayole	–	Charcoal	52	83	140	9
-	–	Donholm	–	Kerosene	180	265	290	5
-	–	Kahawa West	–	Kerosene	155	158	161	4
-	–	Starehe	–	Kerosene	162	181	200	4
-	–	Komarock/kayole	–	Kerosene	160	161	161	3
-	–	Mukuru Kwa Njenga	–	Kerosene	160	173	200	3
-	–	Ruiru Bypass	–	Kerosene	163	165	170	3
-	–	Makadara – Jogoo Road	–	Kerosene	160	163	165	2
-	–	Mukuru kwa Reuben	–	Kerosene	155	158	161	2
-	–	Utawala	–	Kerosene	160	170	180	2
-	–	Thika	–	Kerosene	160	160	160	1
-	–	Starehe	–	LPG	192	228	257	8
-	–	Thika	–	LPG	215	240	257	8
-	–	Kahawa West	–	LPG	169	184	200	6
-	–	Komarock/kayole	–	LPG	183	216	257	6
-	–	Makadara – Jogoo Road	–	LPG	167	194	215	6
-	–	Donholm	–	LPG	219	237	255	4
-	–	Mukuru Kwa Njenga	–	LPG	154	201	283	4
-	–	Ruiru Bypass	–	LPG	207	214	223	4
-	–	Mukuru kwa Reuben	–	LPG	167	187	207	2
-	–	Kamakis	–	LPG	200	200	200	1
-	–	Utawala	–	LPG	231	231	231	1
Vendor type variation								
-	–	–	Petrol station	Kerosene	160	182	285	12
-	–	–	Street vendor	Kerosene	155	184	290	17
-	–	–	Petrol station	LPG	169	226	283	24
-	–	–	Street vendor	LPG	154	204	233	26

**Table A3.** CCT results

Fuel and stove	Test	Mean fuel (g)/power (kWh) consumed	Energy consumed (MJ)	Cost to cook four portions (KSH)	Standard deviation (SD) of fuel (g)/power (kWh) consumed	Calorific value (CV) of fuel (g)/power (kWh) consumed	Mean time taken (mins)	SD of time taken (mins)	CV of time taken
Standard dish tests									
Charcoal	Beans (S)	368.67	11.06	29.12	25.58	7%	68	6	9%
Charcoal	Boiled Water	63.25	1.90	5.00	15.20	24%	12	1	5%
Charcoal	Githeri (S)	386.83	11.61	30.56	52.31	14%	91	11	12%
Charcoal	Rice	77.25	2.32	6.10	8.84	11%	28	4	15%
Charcoal	Sukuma wiki	121.17	3.64	9.57	34.49	28%	23	4	17%
Charcoal	Ugali	156.50	4.70	12.36	26.00	17%	24	2	10%
Charcoal – ICS	Beans (S)	244.17	7.33	19.29	14.63	6%	70	3	4%
Charcoal – ICS	Boiled water	97.00	2.91	7.66	36.51	38%	18	4	24%
Charcoal – ICS	Githeri (S)	231.50	6.95	18.29	26.93	12%	70	2	3%
Charcoal – ICS	Rice	94.17	2.83	7.44	23.75	25%	19	1	6%
Charcoal – ICS	Sukuma wiki	94.33	2.83	7.45	35.85	38%	15	5	30%
Charcoal – ICS	Ugali	109.67	3.29	8.66	22.62	21%	20	7	34%
Electric – EPC	Beans (S)	0.31	1.12	10.23	0.07	24%	57	1	2%
Electric – EPC	Boiled water	0.26	0.95	8.66	0.01	4%	15	1	5%
Electric – EPC	Githeri (S)	0.23	0.83	7.62	0.01	6%	29	8	26%
Electric – EPC	Rice	0.22	0.78	7.12	0.01	6%	21	1	2%
Electric – EPC	Sukuma wiki	0.22	0.78	7.12	0.01	4%	15	1	4%
Electric – EPC	Ugali	0.32	1.16	10.65	0.01	4%	26	2	9%
Electric – IN	Beans (S)	1.46	5.25	48.16	0.02	2%	67	1	2%
Electric – IN	Boiled water	0.23	0.82	7.50	0.00	1%	10	1	6%
Electric – IN	Githeri (S)	1.69	6.09	55.84	0.09	5%	74	4	5%
Electric – IN	Rice	0.40	1.42	13.05	0.08	20%	20	2	8%
Electric – IN	Sukuma wiki	0.28	1.00	9.15	0.04	14%	11	1	6%
Electric – IN	Ugali	0.30	1.07	9.80	0.02	7%	20	2	11%
Electric – IR	Beans (S)	1.43	5.14	47.16	0.06	4%	67	4	6%
Electric – IR	Boiled water	0.27	0.97	8.87	0.01	4%	9	0	3%
Electric – IR	Githeri (S)	1.57	5.65	51.80	0.00	0%	74	2	2%
Electric – IR	Rice	0.40	1.44	13.18	0.02	5%	32	0	0%
Electric – IR	Sukuma wiki	0.38	1.35	12.39	0.03	7%	15	4	24%
Electric – IR	Ugali	0.42	1.50	13.79	0.03	7%	24	6	24%
Ethanol – KOKO	Beans (S)	270.00	7.29	29.27	25.87	10%	65	2	3%
Ethanol – KOKO	Boiled water	61.83	1.67	6.70	0.76	1%	16	1	9%
Ethanol – KOKO	Githeri (S)	318.83	8.61	34.56	27.57	9%	80	8	11%
Ethanol – KOKO	Rice	73.50	1.98	7.97	6.54	9%	25	2	7%
Ethanol – KOKO	Sukuma wiki	60.00	1.62	6.50	5.22	9%	15	1	4%
Ethanol – KOKO	Ugali	60.17	1.62	6.52	2.75	5%	17	1	8%
Ethanol – MS	Beans (S)	270.33	7.30	100.21	9.80	4%	97	3	3%
Ethanol – MS	Boiled water	107.00	2.89	39.66	6.54	6%	29	4	12%
Ethanol – MS	Githeri (S)	219.83	5.94	81.49	37.48	17%	93	1	1%
Ethanol – MS	Rice	99.67	2.69	36.95	1.26	1%	27	0	2%
Ethanol – MS	Sukuma wiki	83.67	2.26	31.02	7.52	9%	26	3	11%
Ethanol – MS	Ugali	94.50	2.55	35.03	2.00	2%	33	6	20%
Kerosene	Beans (S)	123.67	5.29	28.35	23.03	19%	78	17	22%
Kerosene	Boiled water	37.00	1.58	8.48	3.50	9%	16	2	12%
Kerosene	Githeri (S)	173.00	7.40	39.66	6.08	4%	112	8	7%
Kerosene	Rice	47.75	2.04	10.95	4.60	10%	29	0	1%
Kerosene	Sukuma wiki	34.50	1.48	7.91	1.41	4%	24	6	23%
Kerosene	Ugali	38.50	1.65	8.83	7.94	21%	28	5	19%
LPG-1	Beans (S)	152.50	7.02	32.70	7.47	5%	68	2	3%
LPG-1	Boiled water	27.83	1.28	5.97	3.82	14%	13	1	8%
LPG-1	Githeri (S)	185.67	8.54	39.81	2.75	1%	82	12	14%
LPG-1	Rice	40.50	1.86	8.68	10.83	27%	18	1	4%
LPG-1	Sukuma wiki	27.67	1.27	5.93	2.93	11%	14	1	11%
LPG-1	Ugali	47.00	2.16	10.08	6.24	13%	18	1	4%
LPG-2	Beans (S)	135.67	6.24	29.09	2.31	2%	49	0	1%
LPG-2	Boiled water	28.33	1.30	6.07	0.58	2%	10	0	1%
LPG-2	Githeri (S)	183.17	8.43	39.27	2.02	1%	65	2	3%
LPG-2	Rice	38.33	1.76	8.22	4.91	13%	19	1	7%
LPG-2	Sukuma Wiki	35.67	1.64	7.65	3.06	9%	14	0	2%
LPG-2	Ugali	32.00	1.47	6.86	2.12	7%	12	1	5%
Additional tests: hard foods									
Charcoal	Beans	604.50	18.13	47.75	85.99	14%	71	8	11%
Charcoal	Beans (PC)	461.83	13.85	36.48	63.56	14%	78	4	6%
Charcoal	Beans (S)	368.67	11.06	29.12	25.58	7%	68	6	9%
Electric – EPC	Beans	0.37	1.34	12.29	0.02	6%	59	1	2%
Electric – EPC	Beans (S)	0.31	1.11	10.23	0.07	24%	57	1	2%
LPG-2	Beans (PC)	187.50	8.62	40.2	2.78	1%	67	6	9%
LPG-2	Beans (PC) (S)	83.33	3.83	17.86	0.58	1%	30	0	0%
LPG-2	Githeri (PC) (S)	125.67	5.78	26.94	2.52	2%	40	0	0%
LPG-2	Beans	299.50	13.77	64.21	62.05	21%	114	12	11%
LPG-2	Githeri (S)	183.17	8.42	39.27	2.02	1%	65	2	3%
LPG-2	Beans (S)	135.67	6.24	29.08	2.31	2%	49	0	1%
Additional tests: sequential charcoal									
Charcoal	Non-sequential sukuma wiki + ugali	277.67	8.33	21.94	43.20	16%	48	5	10%
Charcoal	Sequential sukuma wiki + ugali	280.33	8.41	22.15	43.28	15%	43	5	12%
